# Ruptured Obturator Internus Muscle Abscess Causing Extensive Retroperitoneal Necrosis

**DOI:** 10.1155/2020/8920178

**Published:** 2020-02-07

**Authors:** Zablon Bett

**Affiliations:** Maseno University School of Medicine, P.O. Box 1777-20200 Kericho, Kenya

## Abstract

Obturator internus muscle (OIM) abscess occurs rarely in adults. Accurate diagnosis is often hindered and delayed due to the deep location of the abscess and the nonspecific clinical features. Even of rarer occurrence is rupture of the obturator internus muscle abscess into the perirectal space and retroperitoneum causing extensive retroperitoneal necrotizing soft tissue infection. We present a case of ruptured left OIM abscess, which initially presented with clinical features, which were suspected as acute pancreatitis. Contrast-enhanced multidetector computed tomography (MDCT) of the abdomen and pelvis revealed ruptured left OIM abscess with extensive fat stranding, fluid collections, and pockets of gas throughout the perirectal space, perisigmoid space, and bilateral posterior pararenal and anterior pararenal spaces as well as thickening of bilateral anterior renal fascia, posterior renal fascia, and lateral conal fascia. These CT findings were consistent with extensive retroperitoneal necrotizing soft tissue infection secondary to ruptured left obturator internus muscle abscess. Broad-spectrum antibiotics were instituted immediately, and the patient was urgently worked up for drainage of the abscess and debridement of the necrotic material. However, the patient's condition deteriorated quickly before the surgical interventions were performed and slipped into septic shock. Emergency resuscitative measures were unsuccessful, and unfortunately, the patient died. The case represents a rare pathology with an unusual presentation, which can be fatal if diagnosis and treatment is delayed.

## 1. Introduction

Obturator internus muscle abscess occurs rarely in adults. Most of the reported cases of obturator internus muscle abscesses affected children [1]. To my knowledge, there are only six reported cases of obturator internus muscle abscesses in adults [2]. Even of rarer occurrence is rupture of the obturator internus muscle abscess into the perirectal space and retroperitoneum causing necrotizing soft tissue infection. The OIM abscess can present with features, which suggest pelvic osteomyelitis or septic arthritis of the hip especially in children [3]. Retroperitoneal necrotizing soft tissue infection has no specific clinical and physical examination features, and therefore, it can be confused for other inflammatory conditions of the abdomen and pelvis, which cause peritonism such as pancreatitis, ruptured appendicitis, and diverticulitis. MDCT of the abdomen and pelvis is the imaging modality of choice for evaluating the retroperitoneum especially when inflammatory conditions are suspected.

## 2. Case Presentation

This is a case of ruptured left obturator internus muscle abscess complicated with extensive retroperitoneal necrotizing soft tissue infection. A 48-year-old male presented to the hospital with diffuse moderate abdominal pain of a 4-day duration, which was of gradual onset. Later, he developed abdominal distension, which had lasted 3 days prior to seeking medical attention. The patient reported normal bowel movements. A day before admission, he started vomiting and also developed hotness of the body. There is no history of trauma in the recent past. His past medical history was relevant for chronic moderate to severe alcohol intake. There is no history of diabetes or hypertension. He was also human immunodeficiency virus (HIV) negative. On the day of admission, he was sick looking and in pain. The blood pressure was 95/62 mmHg, pulse was 98 beats per minute, temperature was 38.2 degrees centigrade, and respiratory rate was 27 breaths per minute. Abdominal examination showed distended abdomen, which was minimally moving with respiration. Diffuse tenderness was present in all the quadrants. Guarding, rigidity, and rebound tenderness were elicited. No shifting dullness was present. No palpable masses were detected. The bowel sounds were present. The hernia orifices were free. Respiratory examination revealed transmitted sounds at both lung bases posteriorly. The cardiovascular examination and central nervous systems were normal.

Laboratory studies revealed leukocytosis (white blood cell count of 16,100/mm^3^), low hemoglobin level of 10.8 g/dl (13.5–17.5 g/dl), raised ESR of 38 mm/hr (1–13 mm/hr), and elevated CRP 154 mg/l (<3.0 mg/l). Liver function tests revealed mild raised gamma-glutamyltransferase 51 U/l (9-48 U/l). Urea and creatinine were normal. Urinalysis was also normal. Initially, he was treated presumptively for acute pancreatitis because of history of chronic alcohol intake and his clinical presentation. However, initial laboratory tests were not supportive of pancreatitis. Serum lipase was 67 U/l (0–160 U/l).

**Figure 1:**
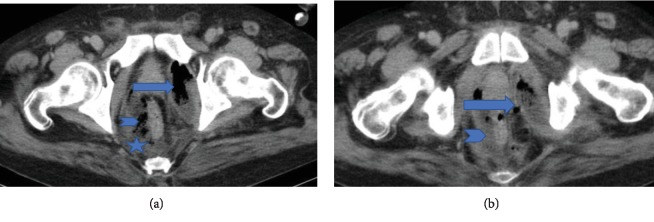
(a) Axial CT scan of the abdomen and pelvis through the hip joints showing abscess (gas and fluid) in the left obturator internus muscle (arrow). Gas (arrowhead) and fat stranding (asterisk) are also seen in perirectal space. (b) Axial CT scan of the abdomen and pelvis at level below the hip joints showing site of the left OIM abscess rupture (arrow). In addition, inflammatory fluid (arrowhead) is seen in perirectal space.

**Figure 2:**
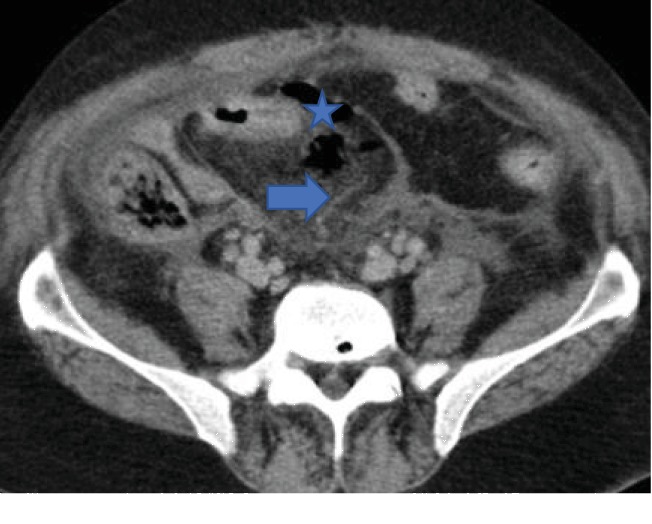
Axial CT image of the abdomen and pelvis showing pericolic gas (asterisk) and fat stranding (arrow).

**Figure 3:**
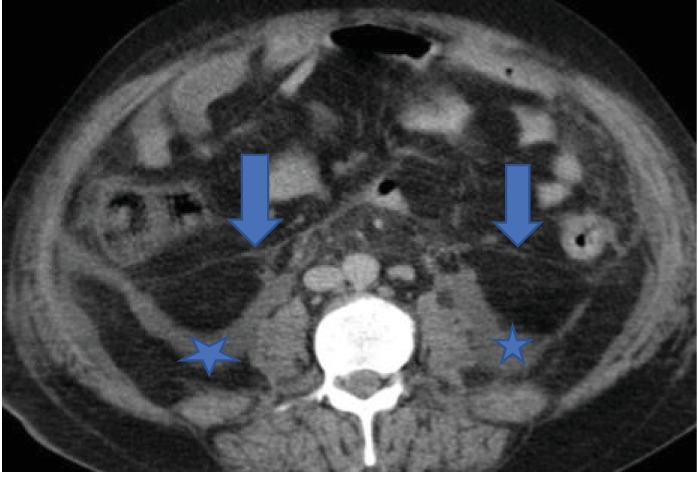
Axial CT image of the abdomen and pelvis at level of L2 showing fluid (asterisk) in bilateral posterior pararenal spaces and thickening of anterior pararenal fascia (arrows).

**Figure 4:**
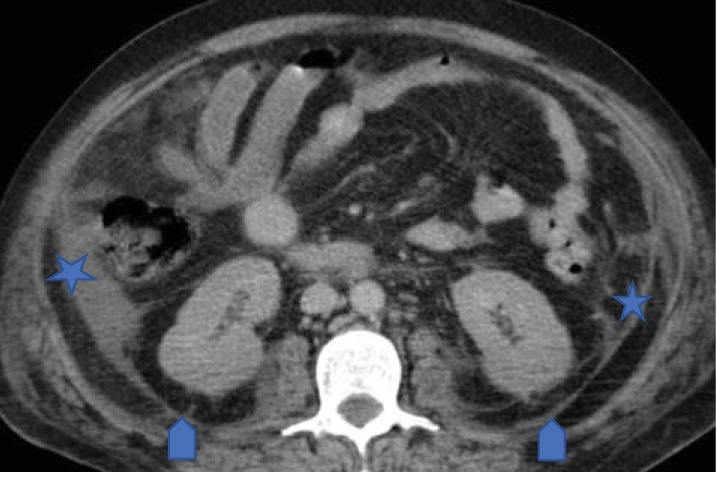
Axial CT image of the abdomen through the level of the kidneys showing thickening of posterior pararenal fascia (arrows) and lateral conal fascia (asterisk).

MDCT scan of his abdomen and pelvis revealed ruptured abscess in the left obturator internus muscle with extension of infective and necrotizing process into the perirectal space (Figures 1(a) and 1(b)). Extensive pockets of gas and fat stranding were also seen in perirectal space extending cephalad to rectosigmoid junction (Figure 2). There was fluid and fat stranding in bilateral pararenal spaces (Figure 3). The anterior renal fascia, posterior renal fascia, and lateral conal fascia were also thickened bilaterally (Figure 4). His liver was diffusely hypoattenuating suggestive of diffuse alcohol-induced steatosis. The appendix was normal. No diverticuli were seen. The iliopsoas muscles were normal. No spine osteomyelitis was detected. The patient was started on broad-spectrum antibiotics (intravenous meropenem and intravenous metronidazole). He was also prepared for drainage of the abscess and debridement of necrotic material. However, within two hours, the patient quickly deteriorated into septic shock. Emergency measures undertaken to stabilize the patient-included aggressive fluid challenge, intubation, central venous catheter insertion, blood pressure support, and administration of vasopressors. Unfortunately, he died while resuscitative measures were being undertaken. Blood cultures which had been taken earlier grew Escherichia coli. Postmortem examination confirmed left ruptured obturator abscess and extensive necrosis in all retroperitoneal compartments.

## 3. Discussion

Obturator internus muscles lie deep in the lateral pelvic walls bilaterally. It arises from inner surface of the obturator membrane lining the obturator foramen and the neighboring surfaces of the pubis and ischium. Medially, it is related to the mesorectal fascia posteriorly and endopelvic fascia anteriorly. It exits the pelvic cavity through lesser sciatic foramen and attaches to greater trochanter [4].

Pyomyositis primarily involves the skeletal muscles and is predominantly caused by bacterial infection. It can manifest diffuse inflammatory process, rapidly progressing necrotic inflammatory disease or as a muscle abscess [5]. Pyomyositis has long been considered a disease of the tropics; however, its incidence has been rising in the temperate countries. The most commonly affected muscles are thigh muscles specifically quadriceps and hamstrings. In the pelvis, the most involved muscles are gluteus and iliopsoas. The obturator internus muscle is rarely involved. Overall, pyomyositis of the obturator internus muscle is more common in children and young adults [6]. Some of the recognized predisposing factors include local trauma, adjacent osteomyelitis, superficial soft tissue infections, and bacteremia. Other associated conditions include alcoholism, HIV, diabetes, and cancer chemotherapy [7].

The bacterial pathogens that are commonly cultured are Staphylococcus aureus and Streptococcus pyogenes. Other pathogens that have been isolated include Escherichia coli, Haemophilus influenzae, Neisseria gonorrhoeae, and Klebsiella pneumoniae [8].

Clinical presentation of obturator internus pyomyositis and abscess is nonspecific because of its deep location and may be confused for septic arthritis of the hip and other inflammatory conditions around the hip joint such as osteomyelitis, transient synovitis, iliopsoas pyomyositis, and among other conditions especially in children [9].

It is important to note that our case did not report any pain or discomfort in the pelvic and hip areas neither was there limitation of his movement. Instead, he presented with marked abdominal symptoms. This is very unusual presentation, which has not been documented according to our knowledge. The CT scan of the abdomen and pelvis revealed obvious abscess in the left obturator internus muscle with rupture defect seen medially. Rupture of the left obturator abscess, lysis of the mesorectal fascia with translocation of the bacteria into the perirectal space, is the most likely cause of severe soft tissue infection in the retroperitoneum in our case. The pathogen isolated was gas forming, and the abscess contained significant amount of gas. Inflammation in the perirectal and perisigmoid spaces also contained numerous pockets of gas, fat stranding, and fluid collections. The disease process diffusely spread throughout the pelvic and abdominal retroperitoneal spaces with resultant fluid collections in pararenal spaces and thickening of posterior renal fascia, anterior renal fascia, and lateral conal fascia. The inflammatory process in this case signifies highly virulent bacterial infection secondary to E. coli in a patient who was alcoholic. This is a picture that is commonly seen in severe acute pancreatitis whereby the inflammatory process involving the released proteolytic enzymes causes fluid collections, fat stranding, and thickening of fascia in the retroperitoneum. In both conditions, MDCT scan of the abdomen and pelvis plays a key role in both diagnosis and assessing the severity and extent of the disease. It is therefore prudent to have a high index of clinical suspicion and order for the MDCT as soon as possible to enable early diagnosis and help in decision-making [10]. Our case was initially suspected to be pancreatitis, but MDCT scan clearly showed a normal enhancing pancreas with no peripancreatic fat stranding. In addition, extensive gas formation is absent in acute pancreatitis unless there is superinfection of necrotic pancreas by gas-forming organisms.

Ruptured appendicitis especially retrocecal can result in retroperitoneal necrotizing fasciitis with fluid collection, fat stranding, and thickening of the fascia in retroperitoneal spaces. It can also cause free pockets of gas in the retroperitoneum [11]. MDCT scan of our case revealed a normal appendix with no inflammatory changes around it.

Another differential diagnosis to consider is iliopsoas abscess; however, in most cases, the iliopsoas abscess is well contained within the muscle. In addition, iliopsoas abscesses especially of tuberculous origin are not associated with gas formation. In the tropics, iliopsoas abscesses are very frequently associated with tuberculous spondylodiscitis [12]. The spine was normal in our case. Our case showed normal psoas muscles.

Ruptured diverticulitis of the colon can potentially cause inflammation of the retroperitoneum with resultant thickening of fascia, fat stranding, and fluid collection [13]. CT scan will reveal the inflamed and ruptured diverticulum in most cases. Our case had no features of diverticulosis.

Once a diagnosis of ruptured obturator internus muscle abscess and necrotizing soft tissue infection is made, the goal of treatment is to stabilize the patient, start broad-spectrum antibiotics, and urgently plan for drainage and debridement. Unfortunately, our patient's conditions worsened so rapidly that surgical and drainage procedures were not undertaken.

## 4. Conclusions

Obturator internus muscle abscess is rare in adults and poses significant diagnostic challenge because of its deep location within the pelvis. The symptoms are often nonspecific and resemble other infective/inflammatory diseases of the hip and pelvic bones. Rupture of the abscess can result in severe retroperitoneal necrotizing soft tissue infection, which mimics inflammatory peritoneal conditions such pancreatitis, ruptured appendix, and diverticulitis. Being a highly fatal condition, a high index of clinical suspicion should be borne so that urgent, appropriate, and aggressive treatment can be instituted at the earliest opportunity. CT of the abdomen and pelvis is the most sensitive diagnostic tool to diagnose and evaluate the disease and also to exclude other differential diagnoses.
